# Health-related quality of life and its associated factors in patients with type 2 diabetes mellitus

**DOI:** 10.1177/2050312120965314

**Published:** 2020-10-26

**Authors:** Forouzan Zare, Hosein Ameri, Farzan Madadizadeh, Mohammad Reza Aghaei

**Affiliations:** 1 Department of Health Technology Assessment, Shahid Sadoughi University of Medical Sciences, Yazd, Iran; 2 Department of Epidemiology and Biostatistics, Shahid Sadoughi University of Medical Sciences, Yazd, Iran; 3 Yazd Diabetic Research Center, Shahid Sadoughi University of Medical Sciences, Yazd, Iran

**Keywords:** Type 2 diabetes mellitus, health-related quality of life, EuroQoL-five-dimension-5 level

## Abstract

**Background:**

Assessing the health-related quality of life in patients with type 2 diabetes mellitus is important for evaluation of treatment outcome. The purpose of this study was to evaluate the health-related quality of life in type 2 diabetes mellitus patients and its related factors in Yazd.

**Methods:**

Data were gathered by using the EuroQoL-five-dimension-5 level instrument as well as using medical records of 734 outpatients with type 2 diabetes mellitus who were referred to the largest governmental diabetes center in South of Iran, Yazd province. When appropriate, the Kruskal–Wallis test or the Wilcoxon test was used to test the difference in the health-related quality-of-life scores in each factor. Finally, the adjusted limited dependent variable mixture model was developed to investigate factors associated with health-related quality-of-life scores.

**Results:**

The mean and median of the EuroQoL-five-dimension-5 level index values of 717 patients who completed the questionnaires were 0.75 ± 0.006 and 0.72 ± 0.20, respectively, and those of the Visual Analogue Scale scores were 69.25 ± 0.63 and 75 ± 30, respectively. The mean scores for health-related quality of life were significantly higher for employed, educated, single, and male patients, as well as patients without comorbidities, diabetes-related complications, and hemoglobin A1c level >7%. Adjusted limited dependent variable mixture model showed that gender, age, marital status, and diabetes-related complications are significant independent predictors of EuroQoL-five-dimension-5 level index value.

**Conclusion:**

The mean scores for health-related quality of life in patients with type 2 diabetes mellitus were moderate in this study, and this finding is consistent with health-related quality-of-life scores reported in other studies conducted in the Middle East region. Therefore, health-related quality of life should be the most important consideration in the management of patients. In parallel, some factors, especially gender, should be considered to improve health-related quality of life.

## Introduction

Type 2 diabetes mellitus (T2DM) is one of the most common chronic disorder conditions around the world. According to the latest report of the International Diabetes Federation (IDF), Iran with more than 5.4 million people with T2DM has third place in the Middle East and North Africa (MENA) region in this regard.^1^ T2DM in both controlled and uncontrolled forms can lead to microvascular (retinopathy, nephropathy, and neuropathy) and macrovascular complications (ischemic heart disease (IHD), stroke, and peripheral vascular disease).^1^ For example, 2.6 million people were visually impaired due to diabetic retinopathy in 2015 and it is projected to rise to 3.2 million in 2020,^2^ and this figure in Iranian diabetic patients was 41.9%.^3^ Another example was diabetic foot ulcer (DFU) with prevalence of 6.3% worldwide and that was 8.1% in Iran.^4^ These figures and complications raise an urgent need to further assess the impact of diabetes on the lives of patients. Health-related quality of life (HRQoL) is a measure for assessing impacts of disease, disorder, or disability on physical, mental, and social domains of health.^5^

The EuroQoL five dimensions (EQ-5D) is the most commonly used form of preference-based instruments for measuring HRQoL, and its use is recommended by the National Institute for Health and Care Excellence (NICE).^6^ The EQ-5D is commonly used in two versions: the EuroQoL-five-dimension-3 level (EQ-5D-3L)^7^ and the EuroQoL-five-dimension-5 level (EQ-5D-5L).^8^ The EQ-5D-3L is the most widely used instrument for assessing HRQoL in clinical and outcome studies.^9^ Nevertheless, a recent review indicated that it suffers from high ceiling effects and insufficient sensitivity in some populations.^10^ In parallel, the EQ-5D-5L was developed as an alternative to the EQ-5D-3L. Similar to the EQ-5D-3L, the EQ-5D-5L describes health in five dimensions but in five levels instead of three levels for each dimension.^8^ The evidence of a systematic review has revealed that the EQ-5D-5L has higher discriminatory power, lower ceiling effects, and better reliability and construct validity than EQ-5D-3L.^10^

It has been recommended to use the EQ-5D-5L for assessing HRQoL in clinical and outcome research.^10^ Only one study has used EQ-5D-5L for patients with T2DM in Iran.^11^ The study used the UK EQ-5D-5L value set that is not appropriate for Iranian patients due to substantial differences between two countries’ populations in terms of health, cultural, and socioeconomic status. According to a report by World Health Organization (WHO), Yazd city, a world heritage city located in center of Iran, has the highest prevalence (14.9%) of T2DM based on hemoglobin A1c (HbA1c) among all cities of Iran.^12,13^ The aim of the present study was to describe the HRQoL of patients with T2DM by Iranian EQ-5D-5L in Yazd province and analyze the impact of socio-demographic and clinical factors on HRQoL.

## Methods

### Study Design and Data collection

This study was carried out on patients with T2DM who were in the waiting list for getting the health services of different wards of the Diabetes Research Center and Clinics in Yazd. This center is one of the largest academic centers in Iran that covers over 10,000 patients with diabetes, and patients are admitted to this center from South part of the country.

Patients completed EQ-5D-5L questionnaire along with a researcher-made questionnaire to capture demographic characteristics of the patients. In addition, clinical data were extracted from medical records of the patients. All patients were invited to a room in the center to take the interview. Patients were selected using a consecutive sampling method between November 2019 and February 2020, and the informed consent was obtained from all patients before participating in the study.

### Study sample

The sample size was calculated based on the mean and standard deviation (SD) of a national survey of patients with T2DM. The national survey assessed the HRQoL by using EQ-5D-3L in a 3472 sample size of Iranian T2DM patients in 2012.^14^ With a mean = 0.70, SD = 0.3, and d = 0.031, a total of 734 patients were selected as the sample size.

### HRQoL

#### EQ-5D-5L instrument

The EQ-5D-5L instrument was developed by the EuroQoL group in 2011 to overcome the limitations of old version of the EQ-5D (i.e. EQ-5D-3L). The EQ-5D-5L includes two parts: a classification system of five dimensions (mobility, self-care, usual activities, pain/discomfort, and anxiety/depression) and a Visual Analogue Scale (VAS). Each of the dimensions of EQ-5D-5L has five levels of response options: no problems, slight problems, moderate problems, severe problems, and extreme problems. The classification system of EQ-5D-5L explains 3125 (i.e. 5^5^) possible health states in total. The VAS is a vertical line with a range of 0 (the worst imaginable health) to 100 (the best imaginable health) that respondents record their health status on it.^8^

Since an Iranian value set for the EQ-5D-3L is available but not for EQ-5D-5L, the crosswalk method introduced by the EuroQoL group was used for generating Iranian interim EQ-5D-5L value set,^15^ and the interim value set has used in two studies in Iran.^16,17^ This method established a connection between the EQ-5D-5L and the EQ-5D-3L descriptive systems, whereas it can generate EQ-5D-5L value set when the EQ-5D-3L value set is available. The EQ-5D-5L instrument was translated into Persian, and its Persian version was confirmed by EuroQoL group (see Supplemental Appendix).^18^ The questionnaires were completed for the patients by the interviewer (F.Z.) in face-to-face interviews, and the informed consent was obtained from all patients before participating in the study. This study was approved by the Committee of Shahid Sadoughi University of Medical Sciences (Approval Number: IR.SSU.SPH.REC.1398.134).

### Statistical analysis

Three of the most widely used basic models for analysis of the EQ-5D utility data are ordinary least squares (OLS), Tobit, and censored least absolute deviation (CLAD).^19^ However, distribution of the data of EQ-5D utility is commonly skewed, multimodal, and is limited at the top and the bottom, and it often has a large number of observations at the top (ceiling effects). Thereafter, using OLS and Tobit is theoretically not the most appropriate model for analysis of these data.^20^ The CLAD model is another alternative that estimates coefficients based on median.^21^ Nevertheless, the CLAD is not appropriate because the most econometric models are based on the mean, and some evidence shows that it performed poorly in studies that had used the EQ-5D instrument as the dependent variable.^22,23^ One of the efforts to overcome these problems is using the estimators based on the beta distribution.

Beta regressions assume that the dependent variable is restricted to a range of values between 0 and 1,^24^ while negative values are observed in the value set of the EQ-5D. Even though beta regressions can cope with the bounded nature of utility data and can reproduce various shapes, multimodality is difficult to capture.^25^ Recently, a new mixture model, called adjusted limited dependent variable mixture model (ALDVMM), was specially developed to deal with the distributional characteristics of EQ-5D instrument.^26^ In the study, the ALDVMM has been adopted to assess the effects of diabetes-related conditions on EQ-5D values.

Since the results of Kolmogorov–Smirnov test (p < 0.05) showed that the distribution of the EQ-5D-5L index values was not normal, Kruskal–Wallis test or Mann–Whitney test was used to test the difference in HRQoL score in each factor. All analyses were performed using STATA version 14.0 for Windows.

## Results

Out of 734 patients, 17 incomplete interviews were excluded from the final analysis, including 7 men. The socio-demographic and clinical characteristics of patients have been described in detail in Tables 1 and 2. The mean and median age of patients was 59.4 ± 20 and 56 ± 13, respectively, and those of body mass index (BMI), waist-to-hip ratio (WHR), duration of diabetics, and HbA1c level were 28.10 ± 0.18 and 27.47 ± 5.83, 0.99 ± 0.01 and 0.97 ± 0.08, 8.65 ± 0.21 and 8 ± 7, 8.67 ± 0.07 and 8.7 ± 2.8, respectively. Among the patients, the most common comorbidities were high cholesterol (11.31%) and chronic obstructive pulmonary disease (COPD; 2.41%).

**Table 1.** Demographic characteristics and EQ-5D-5L index values and EQ-VAS scores (N = 717).

Variable	N (%)	Median EQ-5D-5L index (IQR)	Z	χ^2^ (df)	p value	Median EQ-VAS index (IQR)	Z	χ^2^ (df)	p value
Gender									
Male	425 (59.27)	0.725 (0.201)	2.738		0.0062^a^	75 (25.5)	3.191		0.0014^a^
Female	292 (40.73)	0.710 (0.198)				65 (25)			
Age group, years									
⩽50	198 (27.62)	0.735 (0.198)	−	3.270 (2)	0.195^b^	75 (30)		3.155 (2)	0.206
51–60	276 (38.49)	0.723 (0.202)				75 (25)			
>60	243 (33.89)	0.725 (0.201)				75 (35)			
Education status									
Illiterate/informal^c^	178 (24.83)	0.701 (0.178)	−	8.533 (3)	0.036^b^	75 (30)	–	4.058 (3)	0.255
Primary	260 (36.26)	0.715 (0.17)				70 (25)			
Secondary	227 (31.66)	0.735 (0.201)				70 (25)			
University degree	52 (7.25)	0.736 (0.206)				75 (25)			
Marital status									
Single	16 (2.23)	0.725 (0.157)	−	3.204 (2)	0.205^b^	75 (30)	−	1.351 (2)	0.508
Married	635 (88.56)	0.7115 (0.111)				77.5 (32.5)			
Divorced or widow	66 (9.21)	0.732 (0.271)				70 (15)			
Employment									
Employed	229 (31.94)	0.725 (0.167)	−	4.438 (2)	0.108^b^	75 (20)	−	16.993 (2)	0.0002^b^
Unemployed	242 (33.75)	0.722 (0.201)				65 (25)			
Housewives	246 (34.31)	0.720 (0.201)				65 (25)			

EQ-5D-5L: EuroQoL-five-dimension-5 level. EQ-VAS: EuroQoL Visual Analogue Scale; IQR: interquartile range; df: degrees of freedom.

a Statistical significance of differences calculated using the Mann–Whitney test.

b Statistical significance of differences calculated using the Kruskal–Wallis test.

c They were able to read and write.

**Table 2.** Clinical characteristics and EQ-5D-5L index values and EQ-VAS scores (N = 717).

Variable	N (%)	Median EQ-5D-5L index (IQR)	Z	p value	Median EQ-VAS index (IQR)	z	p
BMI							
<25	187 (26.08)	0.725 (0.211)	0.143	0.8861^a^	75 (30)	0.130	0.8967
⩾25	530 (73.92)	0.725 (0.2)			73 (30)		
WHR							
⩽0.9	77 (10.74)	0.725 (0.198)	0.303	0.7620^a^	75 (30)	0.407	0.6841
>0.9	640 (89.26)	0.725 (0.201)			73 (25)		
Duration of diabetes							
⩽10	497 (69.32)	0.725 (0.157)	0.923	0.356^a^	75 (30)	0.869	0.3846
>10	220 (30.68)	0.715 (0.228)			70 (25)		
Latest HbA1c							
<7	140 (19.53)	0.725 (0.207)	0.377	0.7059^a^	75 (30)	1.782	0.0291
⩾7	577 (80.47)	0.725 (0.157)			71 (25)		
Treatment							
Oral drug	306 (42.68)	0.725 (0.198)	1.119	0.2632^a^	75 (30)	1.380	0.1675
Insulin	411 (57.32)	0.725 (0.201)			70 (30)		
Complications							
Retinopathy (yes)	299 (41.70)	0.708 (0.178)	10.727	0.0000^a^	65 (25)	8.806	0.0000
Retinopathy (no)	418 (58.30)	0.846 (0.302)			75 (20)		
Nephropathy (yes)	183 (25.52)	0.725 (0.201)	4.612	0.0000^a^	65 (25)	10.835	0.0000
Nephropathy (no)	534 (74.48)	0.725 (0.302)			85 (10)		
IHD (yes)	82 (11.44)	0.725 (0.201)	7.351	0.0000^a^	70 (25)	5.184	0.0000
IHD (no)	635 (88.56)	1 (0.186)			77.5 (20)		
Hypertension (yes)	308 (42.96)	0.698 (0.167)	1.509	0.1313^a^	65 (25)	14.148	0.0000
Hypertension (no)	409 (57.04)	0.725 (0.201)			80 (10)		
DFU (yes)	62 (8.65)	0.725 (0.211)	2.515	0.0119^a^	75 (15)	2.781	0.0054
DFU (no)	655 (91.35)	0.813 (0.01)			70 (30)		
Comorbidities							
Yes	318 (44.35)	0.725 (0.200)	3.126	0.0018^a^	70 (30)	0.517	0.6052
No	399 (55.65)	0.725 (0.201)			75 (25)		

EQ-5D-5L: EuroQoL-five-dimension-5 level; EQ-VAS: EuroQoL Visual Analogue Scale; IQR: interquartile range; BMI: body mass index; WHR: waist-to-hip ratio; HbA1c: hemoglobin A1c; IHD: ischemic heart disease; DFU: diabetic foot ulcer.

a Statistical significance of differences calculated using the Mann–Whitney test.

### Distribution of EQ-5D-5L dimensions

**Figure 1.**
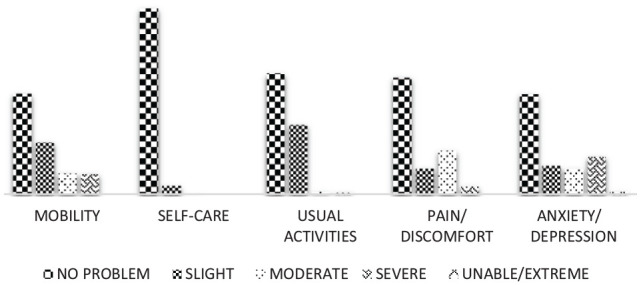
Percentage of problem levels of each EQ-5D-5L dimensions reported by the patients.

Patients’ response for each of the five dimensions of EQ-5D-5L based on the levels is presented in Figure 1. The distribution of responses with no problem (called perfect health state) among the EQ-5D-5L dimensions was 51.74 %, 95.26 %, 61.92%, 59.97%, and 51.46% for mobility, self-care, usual activities, pain/discomfort, and anxiety/depression, respectively. The unable or extreme responses were reported by patients only in the anxiety dimension.

### EQ-5D-5L index values and EuroQoL Visual Analogue Scale scores

The mean and median of EQ-5D-5L index of patients were 0.75 ± 0.006 and 0.72 ± 0.20, respectively, and those of the VAS scores were 69.25 ± 0.63 and 75 ± 30, respectively. The health status conditions observed by EQ-5D-5L index values were almost similar to those observed by VAS scores, and the relationship between EQ-5D-5L index values and VAS scores was good and statistically significant (Spearman correlation test, r = 0.547; p < 0.001).

Table 1 shows that patients with T2DM were more men, age 51 to 60 years old, married, and had primary level of education and most of them were housewives. Also, it presents the association of demographic factors with the EQ-5D-5L index values and VAS scores based on univariate analyses. Regarding the demographic characteristics of the patients, the median of EQ-5D-5L in men was significantly higher (p = 0.006) in comparison to women (0.725 versus 0.710). This difference was also significant for VAS scores (p = 0.0014; 75 versus 65). In addition, the difference between the median scores of the EQ-5D-5L by patients’ education level was significant (p = 0.036), while it was not significant for the median scores of VAS (p = 0.255). The median scores of VAS were statistically significant between different job groups (p = 0.0002), but no difference was detected between groups for the EQ-5D-5L (p = 0.108).

Table 2 shows that patients with T2DM had higher BMI, WHR, and HbA1c level, and presented more retinopathy, nephropathy, IHD, and DFU disorders. Patients with hypertension and comorbidities were lower than half of those in this study. More than half of the patients with T2DM were treated with insulin and had duration of diabetes for less than 10 years.

The results of univariate analyses of the clinical factors showed that it might be associated with the EQ-5D-5L index values, and VAS scores are presented in Table 2. As shown in Table 2, the retinopathy, nephropathy, IHD, DFU, and the total number of comorbidities were significantly associated with EQ-5D-5L index values (p < 0.05), and HbA1c level, retinopathy, nephropathy, IHD, hypertension, and DFU had a significant association with the VAS scores (p < 0.05).

### Regression analyses

Results from the ALDVMM model, where the EQ-5D-5L index values were as a dependent variable and all variables presented in Tables 1 and 2 were as independent variables, showed that age, gender, marital status, treatment status, retinopathy, nephropathy, hypertension, IHD, and DFU were independently associated with EQ-5D-5L index values (p < 0.05), and all relationships were negative (Table 3).

**Table 3.** ALDVMM of EQ-5D-5L index values.

EQ-5D-5L (dependent variable)	Coefficient	SE	z	p	95% CI
Age					
51–60	−0.0097	0.0163	−0.60	0.550	[−0.0417, 0.0222]
>60	−0.0361	0.0181	1.99	0.038	[−0.0046, −0.0666]
Gender					
Female	−0.0598	0.0130	−4.57	0.000	[−0.0341, 0.0855]
Marital status					
Married	−0.1266	0.0430	−2.94	0.003	[−0.2109, −0.0423]
Divorced or widow	−0.0920	0.0239	−3.84	0.000	[−0.1390, −0.0450]
Retinopathy	−0.2052	0.0172	−11.92	0.000	[−0.2390, −0.1715]
Nephropathy	−0.0775	0.0208	3.72	0.000	[−0.0367, −0.1184]
Hypertension	−0.0524	0.0155	3.37	0.001	[−0.0219, −0.0829]
IHD	−0.2057	0.0239	−8.61	0.000	[−0.2525, −0.1588]
DFU	−0.0784	0.0229	−3.42	0.001	[−0.1233, −0.0334]
Constant	1.3511	0.0747	18.07	0.000	[1.2043, 1.4972]

ALDVMM: adjusted limited dependent variable mixture model; EQ-5D-5L: EuroQoL-five-dimension-5 level; SE: standard error; CI: confidence interval; IHD: ischemic heart disease; DFU: diabetic foot ulcer.

## Discussion

One of the important outcomes that is used to evaluate the effects of treatment and management of diabetes in terms of physical and psychosocial functioning is HRQoL. The present study is the first study that assessed the HRQoL based on Iranian interim EQ-5D-5L index values in T2DM outpatients in Iran and the effects of socio-demographic and clinical factors on HRQoL.

The results of our study provided important insights of the HRQoL based on local patients’ characteristics that can be more appropriate in treatment and management of Iranian T2DM outpatients. The mean of the EQ-5D-5L index values and VAS scores was 0.75 and 69.25, respectively. These results were almost similar to the findings reported in a national survey conducted in Iran in 2012, where the mean of Iranian EQ-5D-3L and VAS scores was 0.70 and 56.8, respectively.^14^ The findings are in line with the mean HRQoL score of 61.90 reported by Dehvan et al.,^27^ who systematically analyzed all recent literature on quality of life of Iranian patients with T2DM. The results were also consistent with those reported in other countries of the Middle Eastern region such as Jordan (0.724), and the Riyadh region, Saudi Arabia (0.70).^28,29^ The findings of our country and other countries of the region showed that the mean of the HRQoL scores of patients with T2DM is moderate. When comparing the mean EQ-5D index with the results reported for the diabetes population in countries outside of the region such as China (0.93)^30^ and Vietnam (0.80),^31^ the mean EQ-5D index is lower. This difference may be due to the differences between the health systems of the countries. In addition, the mean EQ-5D-5L index for diabetes patients in this study was lower than human immunodeficiency virus (HIV; 0.80)^32^ but higher skin diseases (0.73),^33^ respiratory diseases (0.66),^34^ dengue fever (0.66),^35^ frail elderly (0.58),^36^ and elderly after fall injury (0.46).^37^

While the mean and median obtained from our study are not consistent with those of Abedini et al.,^11^ who found 0.89 for EQ-5D-5L and 65.22 for VAS in Iran, Abedini et al.’s study is the only study that addressed the HRQoL by using the UK EQ-5D-5L value set based on local data in Birjand region, Iran. Part of this higher HRQoL may be due to difference in value sets used to calculate the EQ-5D-5L in two studies. The UK EQ-5D-5L value set used in Abedini et al.’s study is derived from preferences of general public of a developed country (the United Kingdom) that is different from Iranian general public in terms of demographic and cultural backgrounds. This fact can also be reflected when being compared with the high rate of mean of EQ-5D index value in Korea (0.91), Japan (0.84), Norway (0.85), Australia (0.91), and Germany (0.92).

The percentage of individuals reporting problems in each of the EQ-5D dimensions showed that pain and mobility were the most common problems among subjects. This pattern was similar to those reported recently in a local survey in Birjand, Iran,^11^ and the surveys conducted in Saudi Arabia^38^ and Jordan.^28^

The results of the difference in HRQoL score in each factor and effective factors on HRQoL are discussed in two separate groups: demographic and clinical factors. Among the demographic factors, the mean of the HRQoL scores obtained from EQ-5D and VAS was significantly different in gender, education level, and employment status. Studies carried out on the HRQoL of the patients with T2DM often found that mean HRQoL of male was higher than that of the female patients.^11,14,28,38^ These findings were observed in our study. The main reason for higher HRQoL in men compared to women is the higher level of physical activity in men in developing countries like Iran, as shown in a systematic review on physical activity.^39^ The results of a study showed that T2DM patients with higher level of physical activity reported higher scores on general health (b = 6.66, p < 0.001), vitality (b = 9.05, p < 0.001), social functioning (b = 3.32, p = 0.040), role-emotional (b = 3.08, p = 0.010), and EQ-5D-5L index value (b = 0.022, p = 0.005).^40^ The difference between employment status of men and women in the study can also be the reason for this finding, men more than women were employed (46.9% versus 28.4%). This may lead men to earn more income than women, and subsequently, they had more opportunity for better healthcare. In addition, higher level of education (i.e. university degree) of men than women (13% versus 7%) may explain some of the better HRQoL scores of men. Higher education level increases patients’ perceptions of their disease, and general, psychological, and spiritual QoL.^41^ Another possible explanation for this result is more disease-related worries in women and their less ability to deal with such diseases. In addition, the regression analysis indicated female gender as an independent predictor of poor HRQoL.

Our findings also showed that there is a significant difference between mean EQ-5D scores in patients with different levels of education, as higher level of education was associated with high EQ-5D scores and vice versa. This result was in line with findings reported in other countries, such as Jordan,^28^ Oman,^42^ Indonesia,^43^ and Korea.^44^ Higher EQ-5D score in patients with higher level of education can be explained by them being more aware of the T2DM therapy and the effect of diabetes-related complications, and therefore, it may make them sensitive to their disease and therapy.

In addition, the univariate analysis showed that there is a significant difference between mean scores for VAS scale in three different employment status groups: unemployed, employed, and housewives. The mean VAS scores were higher in employed subjects. This finding is consistent with that of the recent studies evaluating HRQoL of diabetic patients in Iran^11,14^ and Palestine.^45^ This could be due to the fact that employed patients in developing countries have more opportunities to have a better socioeconomic status.

The regression analysis on the effects of the demographic factors on HRQoL showed that, in addition to gender, age and marital status are as significant independent predictors of EQ-5D-5L in this study. There is a negative association between aging and HRQoL among patients with diabetes. Aging has been the most commonly identified negative factor for HRQoL of patients with T2DM in different contexts from developed to developing countries.^29^ It can be explained by the direct relationship between aging and increasing incidence of problems in subjects. In this study, 18% of patients with an age more than 60 years have severe problems in HRQoL compared to 6% of patients aged less than 50 years (data not shown). In addition, the married and divorced patients were linked to lower HRQoL. The reason could be the higher number of women among the married and divorced subjects.

Among the clinical factors, we found that mean scores of HRQoL were significantly different in the latest HbA1c level, comorbidities, and diabetes-related complications in this study. The level of HbA1 was only statistically significant in the mean VAS scores, and it was consistent with that of reported in Birjand region, Iran.^11,46^ In addition, regression analysis showed that among the clinical factors, having comorbidities, retinopathy, nephropathy, hypertension, IHD, and DFU was significantly associated with lower EQ-5D scores. These findings are consistent with those reported in the systematic review carried out by Dehvan et al. on the studies of diabetes and quality of life.^27^ This could explain that each of the complications of diabetes in diabetic patients was associated with severe problems in mobility, self-care, usual activities, pain/discomfort, and anxiety/depression.^11,46^ Interventions including pharmacotherapy, surgery, and educational or lifestyle interventions to control blood glucose or diabetic complications were found to improve quality of life in diabetic patients.^47^

The present study has some limitations that should be noted. First, this study was a cross-sectional questionnaire-based survey, which did not allow us to determine the cause–effect associations between HRQoL and socio-demographic and clinical characteristics. This requires further longitudinal study to understand how the characteristics of patients may affect the HRQoL. Importantly, the participants were restricted to outpatients from one of the largest centers of the Diabetes Research Center and Clinics in Yazd city, center of Iran, who came from South part of Iran. They may not be perfectly representative of other Iranian patients. Thereafter, our results should be applied with caution.

## Conclusion

The present study provides estimates of HRQoL scores for patients with T2DM that can be used in health economic evaluations. Overall, the HRQoL score of patients with T2DM was relatively low in this study, especially among women. The findings are consistent with EQ-5D-5L index values and VAS scores reported for other countries in Middle East region. Identifying strategies to improve the HRQoL in diabetic patients, especially in women, is therefore of particular importance. Our study showed that, in addition to gender, age, marital status, retinopathy, nephropathy, hypertension, IHD, and DFU are as significant independent predictors of EQ-5D-5L in this study.

## Supplemental Material

EQ-5D-5L_English – Supplemental material for Health-related quality of life and its associated factors in patients with type 2 diabetes mellitus Click here for additional data file. Supplemental material, EQ-5D-5L_English for Health-related quality of life and its associated factors in patients with type 2 diabetes mellitus by Forouzan Zare, Hosein Ameri, Farzan Madadizadeh and Mohammad Reza Aghaei in SAGE Open Medicine

EQ-5D-5L_Persian – Supplemental material for Health-related quality of life and its associated factors in patients with type 2 diabetes mellitus Click here for additional data file. Supplemental material, EQ-5D-5L_Persian for Health-related quality of life and its associated factors in patients with type 2 diabetes mellitus by Forouzan Zare, Hosein Ameri, Farzan Madadizadeh and Mohammad Reza Aghaei in SAGE Open Medicine
