# Supplementary material for: Health-related quality of life and its associated factors in patients with type 2 diabetes mellitus
Source: SAGE Open Med. 2020 Oct 26;8:2050312120965314. doi: 10.1177/2050312120965314 (PMC8107944; doi:10.1177/2050312120965314)
Supplement: EQ-5D-5L_English – Supplemental material for Health-related quality of life and its associated factors in patients with type 2 diabetes mellitus [file EQ-5D-5L_English.docx]

|  |
| --- |
| Health Questionnaire |
|  |
|  |
| English version for the UK |
|  |
|  |
|  |

| Under each heading, please tick the ONE box that best describes your health TODAY. | |
| --- | --- |
| **MOBLITY** |  |
| I have no problems in walking about | ❑ |
| I have slight problems in walking about | ❑ |
| I have moderate problems in walking about | ❑ |
| I have severe problems in walking about | ❑ |
| I am unable to walk about | ❑ |
| **SELF-CARE** |  |
| I have no problems washing or dressing myself | ❑ |
| I have slight problems washing or dressing myself | ❑ |
| I have moderate problems washing or dressing myself | ❑ |
| I have severe problems washing or dressing myself | ❑ |
| I am unable to wash or dress myself | ❑ |
| USUAL ACTIVITIES *(e.g. work, study, housework, family or leisure activities)* | |
| I have no problems doing my usual activities | ❑ |
| I have slight problems doing my usual activities | ❑ |
| I have moderate problems doing my usual activities | ❑ |
| I have severe problems doing my usual activities | ❑ |
| I am unable to do my usual activities | ❑ |
| **PAIN / DISCOMFORT** |  |
| I have no pain or discomfort | ❑ |
| I have slight pain or discomfort | ❑ |
| I have moderate pain or discomfort | ❑ |
| I have severe pain or discomfort | ❑ |
| I have extreme pain or discomfort | ❑ |
| **ANXIETY / DEPRESSION** |  |
| I am not anxious or depressed | ❑ |
| I am slightly anxious or depressed | ❑ |
| I am moderately anxious or depressed | ❑ |
| I am severely anxious or depressed | ❑ |
| I am extremely anxious or depressed | ❑ |

The best health
you can imagine

0

5

10

15

20

25

30

35

40

45

50

55

60

65

70

755

80

85

90

95

100

YOUR HEALTH TODY=

- We would like to know how good or bad your health is TODAY.
- This scale is numbered from 0 to 100.
- 100 means the best health you can imagine.

0 means the worst health you can imagine.

- Mark an X on the scale to indicate how your health is TODAY.
- Now, please write the number you marked on the scale in the box below.

The worst health
you can imagine
