# Supplementary material for: Health-related quality of life and its associated factors in patients with type 2 diabetes mellitus
Source: SAGE Open Med. 2020 Oct 26;8:2050312120965314. doi: 10.1177/2050312120965314 (PMC8107944; doi:10.1177/2050312120965314)
Supplement: EQ-5D-5L_Persian – Supplemental material for Health-related quality of life and its associated factors in patients with type 2 diabetes mellitus [file EQ-5D-5L_Persian.docx]

|  |
| --- |
| **پرسشنامه سلامت** |
|  |
|  |
| **نسخه فارسی برای ایران** |
|  |
| ***(Farsi version for Iran)*** |
|  |

| زیر هر عنوان لطفاً مربعی را علامت بزنید که به بهترین نحو وضعیت سلامت **امروز** شما را نشان می‌دهد. | |
| --- | --- |
|  | تحرّک |
| ❑ | هیچ مشکلی در راه رفتن ندارم |
| ❑ | در راه رفتن کمی مشکل دارم |
| ❑ | در راه رفتن نسبتاً مشکل دارم |
| ❑ | **در راه رفتن مشکل جدی دارم** |
| ❑ | **قادر به راه رفتن نیستم** |
|  | مراقبت شخصی |
| ❑ | در شستشوی خودم و یا پوشیدن لباس مشکلی ندارم |
| ❑ | در شستشوی خودم و یا پوشیدن لباس کمی مشکل دارم |
| ❑ | در شستشوی خودم و یا پوشیدن لباس نسبتاً مشکل دارم |
| ❑ | در شستشوی خودم و یا پوشیدن لباس مشکل جدی دارم |
| ❑ | قادر به شستشوی خودم و یا پوشیدن لباس نیستم |
|  | فعالیت‌های عادی **(مانند کار، تحصیل، کارهای خانه، فعالیت‌های خانوادگی یا تفریحی)** |
| ❑ | در انجام فعالیت‌های عادی‌ام هیچ مشکلی ندارم |
| ❑ | در انجام فعالیت‌های عادی‌ام کمی مشکل دارم |
| ❑ | در انجام فعالیت‌های عادی‌ام نسبتاً مشکل دارم |
| ❑ | در انجام فعالیت‌های عادی‌ام مشکل جدی دارم |
| ❑ | قادر به انجام فعالیت‌های عادی‌ام نیستم |
|  | درد/ناراحتیِ جسمی |
| ❑ | هیچ درد یا ناراحتیِ جسمی ندارم |
| ❑ | کمی درد یا ناراحتیِ جسمی دارم |
| ❑ | نسبتاً درد یا ناراحتیِ جسمی دارم |
| ❑ | درد یا ناراحتیِ جسمیِ شدیدی دارم |
| ❑ | بی‌نهایت درد یا ناراحتیِ جسمی دارم |
|  | **اضطراب/افسردگی** |
| ❑ | مضطرب یا افسرده نیستم |
| ❑ | کمی مضطرب یا افسرده هستم |
| ❑ | نسبتاً مضطرب یا افسرده هستم |
| ❑ | به شدت مضطرب یا افسرده هستم |
| ❑ | بی‌نهایت مضطرب یا افسرده هستم |

| - می خواهیم بدانیم وضعیت سلامت **امروز** شما چقدر خوب یا بد است. |
| --- |
| - این مقیاس از 0 تا 100 درجه‌ بندی شده است. |
| - 100 نشان دهنده بهترین وضعیت سلامتی است که می توانید تصور کنید.   0 نشان دهنده بدترین وضعیت سلامتی است که می توانید تصور کنید. |
| - روی این مقیاس با یک X نشان دهید که **امروز** سلامت شما در چه وضعیتی است. |
| - اکنون، لطفاً عددی را که روی مقیاس علامت زده‌ اید، داخل مربع زیر بنویسید. |

بهترین وضعیت سلامتی که می توانید تصور کنید

وضعیت امروز سلامت شما =

بدترین وضعیت سلامتی که می توانید تصور کنید

0

5

10

15

20

25

30

35

40

45

50

55

60

65

70

75

80

85

90

95

100
